# The Penicillin for the Emergency Department Outpatient treatment of CELLulitis (PEDOCELL) trial: update to the study protocol and detailed statistical analysis plan (SAP)

**DOI:** 10.1186/s13063-017-2121-2

**Published:** 2017-08-24

**Authors:** Fiona Boland, Michael Quirke, Brenda Gannon, Sinead Plunkett, John Hayden, John McCourt, Ronan O’Sullivan, Joseph Eustace, Conor Deasy, Abel Wakai

**Affiliations:** 1 Division of Population Health Sciences, Royal College of Surgeons in Ireland (RCSI), 123 St Stephen’s Green, Dublin 2, Ireland; 2 Emergency Care Research Unit (ECRU), Division of Population Health Sciences, RCSI, 123 St Stephen’s Green, Dublin 2, Ireland; 3 Centre for Business and Economics of Health, The University of Queensland, Brisbane, QLD Australia; 4 School of Pharmacy, RCSI, 123 St Stephen’s Green, Dublin 2, Ireland; 5 Clinical Research Centre, RCSI Education and Research Centre, Beaumont Hospital, Beaumont, Dublin 9, Ireland; 6 National Children’s Research Centre, Our Lady’s Children’s Hospital, Crumlin, Ireland; 7 Department of Nephrology, Cardiac-Renal Centre, Cork University Hospital, Cork, Ireland; 8 Department of Emergency Medicine, Cork University Hospital, Wilton, Cork, Ireland; 9 Department of Emergency Medicine, Beaumont Hospital, Dublin 9, Ireland

**Keywords:** Cellulitis, Wound infection, Abscess, Flucloxacillin, Phenoxymethylpenicillin, Randomised controlled trial, Emergency department, Statistical analysis plan

## Abstract

**Background:**

Cellulitis is a painful, potentially serious, infectious process of the dermal and subdermal tissues and represents a significant disease burden. The statistical analysis plan (SAP) for the Penicillin for the Emergency Department Outpatient treatment of CELLulitis (PEDOCELL) trial is described here. The PEDOCELL trial is a multicentre, randomised, parallel-arm, double-blinded, non-inferiority clinical trial comparing the efficacy of flucloxacillin (monotherapy) with combination flucloxacillin/phenoxymethylpenicillin (dual therapy) for the outpatient treatment of cellulitis in the emergency department (ED) setting. To prevent outcome reporting bias, selective reporting and data-driven results, the a priori-defined, detailed SAP is presented here.

**Methods/design:**

Patients will be randomised to either orally administered flucloxacillin 500 mg four times daily and placebo or orally administered 500 mg of flucloxacillin four times daily and phenoxymethylpenicillin 500 mg four times daily. The trial consists of a 7-day intervention period and a 2-week follow-up period. Study measurements will be taken at four specific time points: at patient enrolment, day 2–3 after enrolment and commencing treatment (early clinical response (ECR) visit), day 8–10 after enrolment (end-of-treatment (EOT) visit) and day 14–21 after enrolment (test-of-cure (TOC) visit). The primary outcome measure is investigator-determined clinical response measured at the TOC visit. The secondary outcomes are as follows: lesion size at ECR, clinical treatment failure at each follow-up visit, adherence and persistence of trial patients with orally administered antibiotic therapy at EOT, health-related quality of life (HRQoL) and pharmacoeconomic assessments. The plan for the presentation and comparison of baseline characteristics and outcomes is described in this paper.

**Discussion:**

This trial aims to establish the non-inferiority of orally administered flucloxacillin monotherapy with orally administered flucloxacillin/phenoxymethylpenicillin dual therapy for the ED-directed outpatient treatment of cellulitis. In doing so, this trial will bridge a knowledge gap in this understudied and common condition and will be relevant to clinicians across several different disciplines. The SAP for the PEDOCELL trial was developed a priori in order to minimise analysis bias.

**Trial registration:**

EU Clinical Trials Register (EudraCT number: 2016-001528-69). Registered on 5 April 2016.

ClinicalTrials.gov, ID: NCT02922686. Registered on 9 August 2016.

**Electronic supplementary material:**

The online version of this article (doi:10.1186/s13063-017-2121-2) contains supplementary material, which is available to authorized users.

## Introduction

The Penicillin for the Emergency Department Outpatient treatment of CELLulitis (PEDOCELL) trial is a multicentre, randomised, parallel-arm, double-blinded, non-inferiority clinical trial comparing the efficacy of orally administered flucloxacillin monotherapy with combination orally administered flucloxacillin/phenoxymethylpenicillin for the outpatient treatment of cellulitis in the emergency department (ED) setting.

To prevent outcome reporting bias and data-driven analysis results, the International Conference on Harmonisation of Good Clinical Practice and others have recommended that clinical trials should be analysed according to a pre-specified statistical analysis plan (SAP) [1]. In this paper the SAP that has been finalised prior to any recruitment, and to which all data analyses in the main publication of the trial results will adhere, is described. The PEDOCELL trial has been registered with ClinicalTrials.gov (NCT02922686) and EU Clinical Trials Register (EudraCT number: 2016-001528-69). The SAP was written and submitted without knowledge of the outcome data.

In 2013, the protocol for a randomised controlled trial (RCT) describing a similar intervention (flucloxacillin and phenoxymethylpenicillin versus flucloxacillin and placebo) was published by members of this research group [2]. However, in 2010 and again in 2013, the United States (US) Food and Drug Administration (FDA) recommended a different primary outcome measure for trials of antibiotic treatment for acute bacterial skin and skin structure infections (ABSSSI), which directly impacted on the proposed primary and secondary outcome measures to be utilized in the previous version of this study. In addition, the investigators developed several secondary outcome measures including validation of the Extremity Soft Tissue Infection (ESTI) score, measurement of health-related quality of life (HRQoL) using the EuroQol – 5 dimensions – 5 levels (EQ-5D-5L) instrument, a pharmacoeconomic analysis to assess cost per quality-adjusted life year (QALY) of the new treatment, and an adherence substudy using an electronic medication event monitoring system (MEMS® cap). The investigators have also the created a credible Data and Safety Monitoring Committee (DSMC), a scientific advisory group, the nomination of five site investigators and the recruitment of two full-time research nurses to join the project team. Given the scope and scale of these changes, it was decided that the submission of an entirely new application to the relevant authorities was required. An overview of the current study design is outlined below.

## Trial overview

The PEDOCELL trial is a multicentre, randomised, parallel-arm, non-inferiority trial which will be conducted in five hospitals in the Republic of Ireland (Beaumont Hospital, Connolly Hospital, Cork University Hospital, Mater Misericordiae University Hospital and Mercy University Hospital). It may be necessary to include two contingency sites, dependent on recruitment rates. Those sites are Our Lady of Lourdes Hospital in Drogheda and Midlands Regional Hospital in Tullamore. The trial consists of a 7-day intervention period and a 2-week follow-up period.

Measurements will be undertaken at four specific time points: at the baseline visit, at the early clinical response (ECR) visit on day 2–3 after enrolment, at the end-of-treatment (EOT) visit on day 8–10 post enrolment and at the test-of-cure (TOC) visit on day 14–21 post enrolment.

The study design also includes a substudy to evaluate patient adherence and persistence with therapy using an electronic MEMS® cap. The purpose of this substudy is to describe these parameters in the understudied area of short-course outpatient antibiotic treatment as well as to provide evidence for any adjustments in analysis based on medication-taking behaviour.

## Objectives

The primary objective of the PEDOCELL trial is to conduct a multicentre, randomised, parallel-arm, non-inferiority trial and to determine if orally administered flucloxacillin alone (monotherapy) is non-inferior to orally administered flucloxacillin and phenoxymethylpenicillin (dual therapy) for the ED-directed outpatient treatment of cellulitis.

The secondary objectives of the PEDOCELL trial are:

1. To investigate the relationship between reduction in lesion surface area measured at the ECR visit, and investigator-determined clinical cure measured at the TOC visit. A reduction of ≥ 20% of lesion surface area is the most recently recommended primary outcome measure by the US FDA, while clinical cure is recommended by the European Medicines Agency (EMA)
2. To measure and investigate adherence and persistence of trial patients with outpatient antibiotic therapy measured by self-report and by counting the number of unused study medications at the EOT visit. In addition, to describe adherence and persistence in a substudy using an electronic MEMS**®** cap
3. To perform a within-trial evaluation of the cost per QALY gained from the use of orally administered flucloxacillin compared with combination therapy over a 1-month time horizon from the perspective of the health care payer (direct costs). In a secondary analysis the perspective will be extended to consider costs related to the intervention falling on the patient and the government
4. To externally validate the ESTI score, a HRQoL questionnaire designed to quantify the impact of cellulitis and other ABSSSI on patient HRQoL in clinical trials. Although investigator-determined clinical cure could be considered a composite of objective signs of cure and subjective patient experiences, the ESTI score will allow for quantification of these experiences and the effects of treatment

## Trial procedure

Consecutive patients attending the participating EDs with cellulitis, who meet the inclusion criteria and none of the exclusion criteria for the trial, will be considered eligible for enrolment. Eligibility for enrolment will be determined by a suitably trained member of the research team.

### Inclusion criteria

1. Clinically diagnosed cellulitis, affecting any body part, excluding the perineum, and having any two of the following signs:
  Erythema
  Warmth
  Tenderness/pain of the affected area
  Oedema/induration
  Regional lymphadenopathy
2. Cellulitis deemed treatable with orally administered outpatient antibiotics in which either combination of antibiotic is likely to produce a clinical response (Eron Class 1–2)
3. Written informed consent obtained
4. Sixteen years of age or older
5. Fluency in written and spoken English
6. Willing to return for study follow-up or to have the research nurse visit them for follow-up
7. Willing to receive a telephone call from a study investigator

### Exclusion criteria

1. Penicillin allergy (self-reported or confirmed)
2. Any cellulitis that treating clinicians deem treatable with intravenously administered antibiotics
3. Any cellulitis of the perineal region
4. Patients who have received more than 24 h of effective antibiotics for the current episode of acute cellulitis
5. Any medical condition, based on clinical judgment, that may interfere with interpretation of the primary outcome measures (e.g. chronic skin condition at the cellulitis lesion site)
6. Immunodeficiency from primary or secondary causes (e.g. corticosteroids, chemotherapeutic agents)
7. Previous history of renal dysfunction or known chronic kidney disease under the care of a nephrologist
8. Previous history of liver dysfunction (defined as chronically deranged liver function tests elicited from medical notes or history)
9. Suspected or confirmed septic arthritis
10. Suspected or confirmed osteomyelitis
11. Infection involving prosthetic material
12. Pregnant or lactating women
13. Patients with a previous history of flucloxacillin-associated jaundice/hepatic dysfunction
14. Patients with a previous history of methicillin-resistant *Staphylococcus aureus* (MRSA) colonisation/infection
15. Patients with lactose intolerance diagnosed by a medical professional

### Interventions

Patients will be randomised in a blinded fashion to either orally administered flucloxacillin 500 mg and placebo, or orally administered flucloxacillin 500 mg and phenoxymethylpenicillin 500 mg, both four times daily for 7 days.

### Determination of sample size

The sample size per trial arm was calculated based on an assumed treatment success rate of 85% with orally administered flucloxacillin and phenoxymethylpenicillin, a non-inferiority threshold *Δ* = 12.5% and *α* = 0.025 (as this is a non-inferiority study). Sample sizes were calculated using SAS v9.3 (SAS Institute Inc., Cary, NC, USA). Given the preferred study power of 90% for non-inferiority trials and a clinical evaluability rate of 80%, it is estimated that a minimum sample size of 207 in each treatment arm is required (*n* = 414).

The non-inferiority margin was based on a combination of statistical reasoning and clinical judgement. The non-inferiority margin of 12.5% was considered clinically acceptable based on best current evidence [3]. Additionally, the Infectious Diseases Society of America has recommended a non-inferiority margin of between 10 and 15% for RCTs of antibiotic treatment for cellulitis [4]. Furthermore, current RCT evidence indicates that the efficacy of the comparator intervention (i.e. dual therapy with flucloxacillin and phenoxymethylpenicillin) is 83–87% efficacious. Our sample size for each trial arm was calculated based on an assumed treatment success rate of 85%, which is the midpoint of this 83–87% success range.

### Randomisation and blinding

Patients will be stratified by site and type of infection and allocation will be blocked using random permuted blocks of varying size of 2, 4 and 6. A person independent of the trial will create a randomisation plan in advance of the treatment packets being made up. This plan will contain a list of packet numbers and whether the packet number corresponds to study arm A or B. The treatment packet of study medications will be made up by a pharmacist before commencement of the study. Following this, all packets will be assigned a label by the pharmacist corresponding to the packet numbers above. The pharmacist, in addition to the outcome assessors and patients, will be blinded as to which patient receives which treatment pack.

A password-controlled Excel file will be setup (by a person independent of the trial). A research nurse will enter the patient identification number in the Excel file and the packet number to be given to the patient will be revealed. The user of the Excel file is blinded as to which arm of the study the patient is assigned to. The password-controlled Excel file will have built-in validation ensuring that patient identification numbers are entered in strict sequential order and cannot be duplicated or skipped in error.

Individual, sealed patient-number envelopes will be kept in a secure locked press for emergency unblinding access. Contact numbers of the chief investigator (AW) and another study investigator (MQ) will also be available in the circumstances when unblinding is required.

The DSMC may be unblinded to individual study treatment assignments, as needed, to adequately assess safety issues.

### Outcome measures/endpoints

#### Primary outcome measure

The primary outcome measure is investigator-determined clinical response measured at the TOC visit. Measurement of this outcome will be as follows:

1. A suitably trained member of the study team will determine clinical cure at the TOC visit. This is a clinically determined response to treatment based on the judgement of a suitably trained member of the study team
2. Definition of clinical cure:
  - No treatment failure at any previous visit
  - Resolution or minimal presence of the following signs and symptoms from the baseline assessment based on the study investigator’s clinical assessment:
    1. Erythema
    2. Swelling
    3. Tenderness
    4. Induration

Follow-up assessments will be in person. In the unlikely event that this is not possible, the research nurse will contact the patient by telephone and obtain as much follow-up information from the patient as possible.

#### Secondary outcome measures

The principal secondary outcome measure is a ≥ 20% reduction in lesion surface area on day 2–3 after enrolment compared to the baseline visit. In addition, the following secondary outcome measures will be assessed:

- Clinical treatment failure at each follow-up visit
- Adherence and persistence of trial patients with outpatient antibiotic therapy at EOT (day 8–10 post enrolment)
- HRQoL assessments at each follow-up visit
- A pharmacoeconomic assessment of cost per QALY

##### 1. ECR measured on day 2–3

ECR measured on day 2–3 is defined as ≥ 20% reduction in the lesion surface area from that which was measured at enrolment. Objective measurement of lesion size is the US FDA-recommended outcome measure of choice. In order to enhance the validity of the study findings, an objective measure of the percentage decrease in the diameter of infection at day 2–3 will be utilized as a secondary outcome measure. Surface area measurement will be achieved by multiplying the vertical and horizontal diameter of the area of lesion.

##### 2. Clinical treatment failure

Any patient outcome designated as a clinical treatment failure at any time before and including the TOC visit will be categorised as a treatment failure.

##### 3. Adherence and persistence of trial patients with outpatient antibiotic therapy

Medication adherence will be measured at EOT in various ways:

1. By self-report
  - Have you taken all of your antibiotic doses as prescribed? (yes/no)
  - If not, how many times would you estimate you did not take your doses as prescribed?
  - Did you finish your antibiotic course?
  - If not, when did you stop? (day 0–7)
2. By counting the number of unused study medications at the EOT visit. Patients will be asked to bring their medication with them to the EOT visit so that a pill count can also be performed
3. By using an electronic monitoring system (MEMS® cap). The MEMS**®** cap will be used to measure adherence in a subset of 100 patients assigned to either treatment arm at the Beaumont Hospital study site only. The MEMS® cap will be fitted to the dispensed medication bottle. Patients will be informed that the cap will monitor each opening of the bottle and, as such, they should only open the bottle when they are about to ingest a dose. Any other openings should be recorded and the research team should be informed. The MEMS® cap should be returned with the clinical trials supplies on the follow-up visits

Medication persistence may be defined as the duration of time from initiation to discontinuation of therapy [5]. Persistence will be measured using the MEMS® cap in the subgroup of 100 patients. It will be measured by determining from the MEMS® cap report the time (in days) from the start day of treatment to the day of the last dose taken without regard for any missing doses or days in between. Missing doses and days will be described separately in descriptions of adherence.

##### 4. HRQoL for economic evaluation

The Foundation for the National Institutes of Health Biomarkers Consortium Project Team and the FDA have explicitly indicated that patient-reported outcome measurements, or how a patient “feels and functions,” are important, required by regulation, and should be measured at early and late time points. However, no RCT included in the Cochrane review of cellulitis treatment assessed HRQoL [6]. The planned study aims to measure HRQoL using the EQ-5D-5L instrument and also to validate a novel disease-specific HRQoL instrument, the ESTI score, in order to address these knowledge gaps. Validation of the ESTI score may also inform future health care decisions about more costly interventions for the treatment of cellulitis. In the meantime, a large proportion of the economic cost of skin and soft tissue infections (SSTIs) caused by community-acquired methicillin-resistant *Staphylococcus aureus* (CA-MRSA) stems from losses in patient productivity [7]. However, the economic impact of treating ABSSSI for patients presenting to EDs is unknown; by performing a pharmacoeconomic analysis we will provide useful insights regarding the economic impact of treating this relatively common ED condition.

The EQ-5D-5L has been selected as the most appropriate measure of HRQoL. It is a standard measure of self-reported HRQoL which includes five domains: mobility, self-care, usual activities, pain/discomfort and anxiety/depression. The ESTI score will also be measured and mapped against the ED-5D-5 L as a robustness check and this outcome will be used as an alternative way to measure QALYs. The EQ-5D-5L, the 12-Item Short Form Health Survey (SF-12) and ESTI score will be used to obtain patient reports of HRQoL and then used in the estimation of QALYs. The (SF-12) instrument is routinely used as a sensitivity check for quality of life within trials.

##### 5. Pharmacoeconomic analysis of cost per QALY

Although the cost of treating cellulitis may be relatively low, it is important to understand the full health outcomes associated with acute cellulitis in order to establish complete cost-effectiveness for resource allocation decisions. We will perform a within-trial economic evaluation of the cost per QALY gained from the use of orally administered flucloxacillin alone compared with using combination therapy of orally administered flucloxacillin and phenoxymethylpenicillin over a 1-month time horizon from the perspective of the health care payer (direct costs). The QALY measure captures the impact of a treatment on a patient’s length of life and also the impact on their HRQoL and is widely used in health economics as a summary measure of health outcome, which can inform health care resource allocation decisions.

In a secondary analysis the perspective will be extended to consider costs related to the intervention falling on the patient and the government. Costs will be estimated from recorded resources used during the trial, and from health care utilisation and absenteeism/productivity (for the broader perspective) data collected from patients using questionnaires administered at all time points. The EQ-5D-5L, (SF-12) and ESTI score will be used to obtain patient reports of HRQoL and used in the estimation of QALYs. The health economics study aims to identify the within-study incremental cost-effectiveness ratio (ICER; i.e. the costs and benefits of the treatment compared to the control group). Cost per QALY will be the main outcome derived.

### Data collection

An electronic Case Report Form (eCRF) will be used to collect all data. For a paper version of this CRF (paper CRF or pCRF; see Additional file 1). Briefly, in addition to the outcome measures specified above, demographics, including age, gender (male/female), ethnicity (White/Black/Asian/Other) and current smoker (yes/no), vital signs including blood pressure, heart rate, temperature, level of alertness, and previous medical history (including history of cardiac disease, diabetes, vascular disease, venous insufficiency, any other diseases) and Charlson Comorbidity Index (CCI) will be collected.

Data on health resource use costs and quality of life will also be collected (see Additional files 2 and 3). The latter will be used to calculate QALYs for the pharmacoeconomic analysis. The CRFs will include a health resource use questionnaire that will be adapted from the standard Client Service Receipt Inventory (CSRI) that is used in trials to collect information about extra resource utilization (e.g. general practitioner visits), medication use, work status and payment of health costs. Patients will receive this at each time point in the trial. Unit costs will be retrieved from Irish and United Kingdom (UK) reference costs. Medication costs will be taken from the Health Service Executive (HSE) Primary Care Reimbursement Service database. The HSE is the statutory provider responsible for all the public health and social services in hospitals and in the community in the Republic of Ireland. Life years gained will be extracted from the published literature. Costs will also include treatment costs. The costs to patients, in terms of time input and travel expenses over the course of the trial, will be estimated. These costs will reflect real-life expenditure post trial. There are still no reference costs available in Ireland (similar to the National Health Service unit costs and Personal Social Services Research Unit costs in the UK), so we will collect unit cost data for relevant resource items from local data sources and expert opinion, or from similar utilisation in the UK if none are available in Ireland. All costs will be up-rated to 2017 prices using the health component of the consumer price index in Ireland. Pounds will be converted to euros using the 2017 exchange rate from the Central Bank of Ireland.

All data being entered into the eCRF will be validated according to agreed criteria appropriate to the data type (i.e. upper and lower limits on numeric values and dates, drop-down lists of valid responses or radio-button selection for multiple option items, dependency-driven data items will only be collected where necessary and relevant).

### Analyses sets/populations/subgroups

For the analysis of the primary outcome both intention-to-treat (ITT) and per-protocol (PP) analyses will be performed. Initially, ITT analyses will be performed and will include all patients randomised to the trial regardless of whether they have taken the study drug or not. PP analysis will be defined as in a previous randomised controlled trial for uncomplicated skin abscesses conducted by Talan et al. in the United States [8]. It will include participants who either took ≥ 75% of the total doses of study drug or placebo during the first 5 days and had an in-person test-of-cure visit or were determined to have had clinical failure before the test-of-cure visit and received ≥ 75% of the doses provided during the first 48 h of the treatment period. In a non-inferiority trial setting it is suggested that a PP analysis may be more appropriate than ITT since it is more likely to reflect actual differences between the two treatments [9]. Furthermore, ITT analysis may be interpreted as being too liberal in a non-inferiority trial and may bias toward making the two treatments appear similar. As a result, both an ITT and PP analysis will be performed on the resulting data to assess non-inferiority. Non-inferiority will only be declared if both ITT and PP analysis support non-inferiority.

### Handling of missing data values

Any missing data or data anomalies will be communicated to the study site(s) for prompt clarification and resolution. It is not anticipated that there will be a huge amount of missing data. However, in the unlikely event that there is more than 10% of data values missing, missing values will be imputed, if possible, using a suitable imputation method.

## Description of statistical methods

Data analysis and reporting will proceed according to the Consolidated Standards of Reporting Trials (CONSORT) extension to non-inferiority and equivalence RCTs [10]. The trial statistician will be blinded to the group status.

### Trial profile, recruitment and retention

The flow of trial patients will be displayed in a CONSORT diagram [11]. The number of screened patients who fulfilled trial inclusion criteria, and the number included in the primary and secondary analyses, as well as all reasons for exclusions in primary and secondary analyses, will be reported in addition to those withdrawing consent and those lost to follow-up.

### Demographic and baseline characteristics

The first stage of analysis will be to use appropriate descriptive statistics to describe recruited individuals, and to investigate comparability of the trial arms at baseline. For categorical measures the number of patients and percentage will be presented, and for continuous scales the mean and standard deviation (SD) will be presented. For continuous scales which show evidence of, or are expected to show some skew, a median and interquartile range may also be presented, or substituted for the mean and SD.

### Primary endpoint

Non-inferiority of the primary outcome measure, the percentage of patients cured on day 14–21, will be assessed by calculating a two-sided 95% confidence interval (CI) on the difference of proportions between the two trial arms. Only one side of the confidence interval is of interest; this equates to a one-sided test with alpha at 0.05/2 (i.e. 0.025). If the upper limit of the CI is less than the non-inferiority margin threshold of 12.5%, then non-inferiority will be inferred.

Secondary analyses will investigate the effects of further adjustment for any variables displaying marked imbalance between the arms at baseline.

### Secondary endpoints

1. ECR measured on day 2–3 post enrolment
  - The percentage of patients in each trial arm cured on day 2–3 will be assessed similar to the primary endpoint by calculating a two-sided 95% confidence interval (CI) on the difference of proportions between the two trial arms
  - The relationship between clinical measurements at ECR and TOC will also be investigated
2. Clinical treatment failure
  A participant with clinical treatment failure for the duration of the study will be censored and the distributions of the two trial arms of the trial will be compared using the logrank test
3. Adherence and persistence of trial patients with antibiotic therapy
  Initially, appropriate descriptive statistics will be used to describe all measures of adherence. More specifically, the following will be calculated and compared between the two arms of the trial using appropriate statistical tests (e.g. two-proportions z-test, chi-squared test, Fisher’s exact test):
  - The proportion of patients who took all doses as measured by self-report, pill count and MEMS® cap
  - The proportion of patients who took more than 80% of doses as measured by self-report, pill count and MEMS® cap
  - Using the MEMS® cap, the proportion of patient’s who took exactly four doses a day for the duration of the treatment
  - Using the MEMS® cap to investigate dosage interval adherence by calculating the following:
    (i) The proportion of patients who took all doses within 2 h of the recommended 6-hourly interval
    (ii) The proportion of patients who took all doses within 4 h of the recommended 6-hourly interval
  - Using the MEMS® cap, a frequency table of number of days to discontinuation
  - Using the MEMS® cap only, a frequency table of various adherence levels (0–10%, 11–20%, 21–30%, etc.), ignoring timing interval, and the corresponding proportion of patients with clinical response will be constructed

Additionally, regression analysis will be used to investigate determinants of good adherence (defined as > 80% adherence) for each measure of adherence (self-report, pill count and MEMS® cap). Variables will include age, gender, ethnicity, disease severity, number of concomitant medicines, health care utilisation and the incidence of adverse drug reactions.

4. HRQoL assessments
  HRQoL scores will be derived from the EQ-5D-5L, the (SF-12) and the ESTI score mapped onto the EQ-5D-5L. The ESTI score is a disease-specific HRQoL instrument developed to quantify patients’ subjective experiences associated with having an ESTI [12]. However, it has not been externally validated or used as an outcome measure for HRQoL assessment in a clinical trial evaluating different management strategies for ESTI. The objective of this substudy is to externally validate the ESTI score and use it as an RCT outcome measure. The feasibility and practicality of the ESTI score will be examined by looking at the response rate and the proportion of missing responses at all time points. Correlations between ESTI score responses and the relevant questions in the EQ-5D-5L and correspondence between overall scores obtained on the ESTI score and the EQ-5D-5L will be examined to assess the ability of the ESTI score to measure what is intended. The distribution of health states and the mean ESTI scores for all time points will be examined to see if the ESTI score differentiates between health states. One would expect the ESTI score to detect important changes in health and an improvement in the scores with time. This responsiveness or sensitivity to clinical change will be assessed by the mean change in score for a scale divided by the SD of the change. Values greater than or equal to 0.2, 0.5 and 0.8 indicate small, moderate and large clinical changes, respectively. This standardised method will allow comparisons to be made between the ESTI score and the EQ-5D-5L. Additionally, to examine if the ESTI score detects important changes in health, discriminant validity will be used. Briefly, discriminant validity will involve determining if using dichotomous outcome measures (20% or greater reduction in cellulitis lesion, yes/no; clinical treatment failure, yes/no; clinical relapse, yes/no; compliance with therapy, yes/no; and adverse events; yes/no) as a method of splitting the sample to determine if the ESTI score can detect a difference between groups. Linear regression analyses will also be used to develop a mapping relationship between the ESTI score and the EQ-5D-5L. The purpose of the mapping is to predict health utilities in other data sets and hence the accuracy of the model’s predictions will be assessed. Both the lowest and highest quartiles will be evaluated separately to see if errors are affected by the severity of the cellulitis
5. Pharmacoeconomic analysis
  HRQoL scores will be multiplied by the duration of time spent in each health state, in order to generate QALYs. The area under the curve (AUC) approach will be used for estimating QALYs, assuming a linear transition between each follow-up time point. The AUC will be 21 days with time point at each follow-up having a utility score. Differences in mean costs and QALYs between the two arms of the trial will be estimated, accounting for differences in patient characteristics and baseline costs and QALYs. If one strategy is not dominant (less costly and more effective), the incremental cost effectiveness ratios (ICER) of the cost per QALY gained will be calculated and bootstrapped estimates (with replacement, 1000 estimates) of the sampling distribution of costs will be generated to explore the uncertainty around the ICER. A cost-effectiveness acceptability curve will be used to describe the probability that the cost per QALY gained from the within-trial analysis is cost-effective for a range of levels of willingness to pay the decision-maker (their ceiling cost-effectiveness ratio). The net benefit will be estimated at a level of willingness to pay from €20,000 to €45,000 (National Centre for Pharmacoeconomics). The calculation of QALYs will be conducted, using a measure of quality of life. In the analysis, the non-parametric bootstrap method will be used to produce a within-trial probabilistic sensitivity analysis of the ICER. A scatterplot of the cost-effectiveness plane, the 95% cost-effectiveness ellipse and cost-effectiveness acceptability curve will be presented.

### Sensitivity analyses

If in the unlikely event that a research nurse has to contact the patient by telephone to obtain follow-up assessments, a sensitivity analysis excluding all telephone follow-up assessments will be conducted.

## Protocol and SAP deviations and violations

All substantial protocol and SAP violations or deviations from the initial design and methods of the trial will be listed.

## Discussion

This paper presents the detailed SAP for the PEDOCELL trial in order to avoid risks of outcome reporting bias and data-driven results. Of the pre-specified results from the trial, we plan to report the primary and some of the secondary outcome measures in separate publications. Due to the complexity of the adherence and persistence of trial patients with antibiotic therapy and HRQoL assessments these will be reported in separate publications.

## Current trial status

Ethical approval was granted by the Clinical Research Ethics Committee, University College Cork on 23 September 2016; the PEDOCELL trial has been registered with ClinicalTrials.gov (NCT02922686) and its protocol has been published [2]. Recruitment is due to start in August 2017.

## Additional files

Additional file 1: Case Report Form (CRF). Contains a paper version of the electronic case report form (eCRF) which will be used in the PEDOCELL trial. (DOCX 445 kb)

Additional file 2: Health-related quality of life (HRQoL) questionnaires. The HRQoL questionnaires to be used in the PEDOCELL trial are to be found in Additional File 2. The EQ-5D-5L, the SF-12 and the Extremity Soft Tissue Infection (ESTI) score will be used to measure HRQoL outcomes in patients enrolled to the PEDOCELL trial at each follow-up visit. (DOCX 86 kb)

Additional file 3: Health resource use questionnaires. The health resource use questionnaires will be completed at the baseline visit, early clinical response visit, end of treatment visit, test of cure visit and any unscheduled visit. The results of these questionnaires will contribute to the pharmacoeconomic analysis for the PEDOCELL trial. (DOCX 123 kb)

## Abbreviations

ABSSSI: Acute bacterial skin and skin structure infections
AUC: Area under the curve
CA-MRSA: Community-acquired methicillin-resistant *Staphylococcus aureus*
CI: Confidence interval
CONSORT: Consolidated Standards of Reporting Trials
CRF: Case Record Form
DSMC: Data Safety and Monitoring Committee
ECR: Early clinical response
eCRF: electronic Case Record Form
ED: Emergency department
EMA: European Medicines Agency
EOT: End-of-treatment
EQ-5D-5L: EuroQol – 5 Dimensions – 5 Levels
ESTI: Extremity Soft Tissue Infection
FDA: Food and Drug Administration
HRQL: Health-related Quality-of-Life
ICER: Incremental cost-effectiveness ratio
ITT: Intention-to- treat
MEMS®: Medication Event Monitoring System
PEDOCELL: Penicillin for the Emergency Department Outpatient treatment of CELLulitis
PP: Per protocol
QALY: Quality-adjusted life year
RCSI: Royal College of Surgeons in Ireland
RCT: Randomised controlled trial
SAP: Statistical analysis plan
SD: Standard deviation
(SF-12): 12-Item Short Form Health Survey
SSTI: Skin and Soft Tissue Infections
TOC: Test-of-cure
UK: United Kingdom
US: United States
