# Supplementary material for: The Penicillin for the Emergency Department Outpatient treatment of CELLulitis (PEDOCELL) trial: update to the study protocol and detailed statistical analysis plan (SAP)
Source: Trials. 2017 Aug 24;18:391. doi: 10.1186/s13063-017-2121-2 (PMC5571617; doi:10.1186/s13063-017-2121-2)
Supplement: Supplementary file 1 — Case Report Form (CRF). Contains a paper version of the electronic case report form (eCRF) which will be used in the PEDOCELL trial. (DOCX 445 kb) [file 13063_2017_2121_MOESM1_ESM.docx]

Case Report form

**PEDOCELL**

**Confidential**

**SITE NAME Beaumont Hospital SITE CODE 01**

**SITE I.D**  **PATIENT ID**

| **VISIT** | **DATE (*dd/mm/yyyy*)** | **TIME (24hour)** |
| --- | --- | --- |
| **Baseline** (Day 0) | / / |  |
| **Early Clinical Response** (Day 2-3) | / / |  |
| **End Of Treatment** (Day 8-10) | / / |  |
| **Test Of Cure** (Day 14-21) | / / |  |

## Withdrawal of Participant Yes*

Loss to Follow up Yes *

## **Reason*__________________________________________________________________


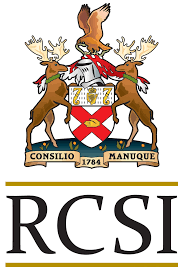


**BASELINE VISIT**

**CASE Report FORM**

**confidential**

| **PARTICIPANT detailS** | |
| --- | --- |
| Home Phone Number |  |
| Mobile Number |  |
| Patient I.D |  |
| Site I.D |  |

### **PART A INFORMED CONSENT**

### **Inclusion** criteria

- Select “Yes” or “No” for each criteria
- **Note:** if any of the **INCLUSION** criteria is marked **NO** the participant is **NOT** eligible for inclusion in the trial and must **NOT** be included in the study**.**

| **INCLUSION CRITERIA: ALL ANSWERS MUST BE *YES*** | | |
| --- | --- | --- |
| 1 Clinically diagnosed cellulitis, wound infection or abscess affecting any body part, **excluding** the perineum, and having any 2 of the following signs:   1. Erythema 2. Warmth 3. Tenderness/Pain of affected area 4. Oedema /Induration 5. Regional lymphadenopathy 6. Purulent drainage | Yes | No |
| 2. Cellulitis, wound infection or abscess deemed treatable with oral antibiotics in which either combination of antibiotics is likely to produce a clinical response | Yes | No |
| 3. Written informed consent obtained | Yes | No |
| 4. 16 years of age or older | Yes | No |
| 5. Fluency in written and spoken English | Yes | No |
| 6. Willing to return for study follow-up or to have the research nurse visit them for follow-up | Yes | No |
| 7. Willing to receive a telephone call from a study investigator | Yes | No |

### **exclusion** criteria

- Select “Yes” or “No” for each criteria.
- **Note:** If any of the **exclusion** criteria is marked “**YES”** the participant is **NOT** eligible for the inclusion in the trial and must **NOT** be included in the study.

| **EXCLUSION CRITERIA:** ALL ANSWERS MUST BE ***NO*** | | |
| --- | --- | --- |
| Penicillin allergy (self-reported or confirmed) | Yes | No |
| Any cellulitis, wound infection or abscess that the treating clinician deems treatable with intravenous (IV) antibiotics | Yes | No |
| Any cellulitis, wound infection or abscess of the perineal region | Yes | No |
| Patients who have received more than 24 hours of effective antibiotics for the current episode of acute cellulitis, wound infection or abscess | Yes | No |
| Any medical condition, based on clinical judgment that may interfere with interpretation of the primary outcome measures (e.g chronic skin condition lesion site) | Yes | No |
| Immunodeficiency from primary or secondary causes (e.g corticosteroids, chemotherapeutic agents). | Yes | No |
| Previous history of renal dysfunction or known chronic kidney disease under the care of a nephrologist | Yes | No |
| Previous history of liver dysfunction (defined as chronically deranged liver functions test elicited from medical notes or history) | Yes | No |
| Suspected or confirmed septic arthritis. | Yes | No |
| Suspected or confirmed osteomyelitis. | Yes | No |
| Infection involving prosthetic material. | Yes | No |
| Pregnant or lactating women. | Yes | No |
| Patients with a previous history of flucloxacillin- associated jaundice/hepatic dysfunction. | Yes | No |
| Patients with a previous history of MRSA colonisation/infection. | Yes | No |
| Patients with lactose intolerance diagnosed by a medical professional. | Yes | No |

#### **Section 1.0 Informed Consent**

|  | **Date Consent Obtained** |
| --- | --- |
| Informed consent | **____/_____/_____** |
| MEMS® cap study consent | **____/_____/_____** |

Name of person taking informed consent (BLOCK CAPITALS)______________________________________

#### **Section 1.1 HRQL and Health Resource Use Questionnaires**

Has the patient completed the questionnaires? *If no state reason in comment section*

1. EQ-5D-5L Yes No
2. SF 12 Yes No
3. ESTI-Score Yes No
4. Health Resource Use Questionnaire Yes No

Comment_________________________________________________________________________________

#### **Section 1.2 Demographic History**

1. Referral : Self-referral GP referral

2. Gender: Male Female

3. Date of birth **__ __ / __ __ / __ __ __ __** (dd/mm/yyyy)

4. Race:

*Indicate “Yes” or “No” for each, or if race not listed document in other/comment.*

White Yes No

Black Yes No

Asian Yes No

Other/Comment____________________________________________________________

5. BMI: Weight: ________ kg Height: _______ cm

6. Smoking History**:** Current Smoker Yes No

7. Alcohol Intake (*refer to table below*): Non-drinker Estimate Units per week **______**

| 1unit | A pub measure of spirits (35.5ml) |
| --- | --- |
| 1 unit | A small glass of wine (12.5% volume) |
| 1 unit | A half pint of normal beer |
| 1 unit | An alcopop (275ml bottle) |

| **Section 1.3 Relevant Medical History None** | | | | | |
| --- | --- | --- | --- | --- | --- |
| **1** | | 5 |  | | |
| **2** | | 6 |  | | |
| **3** | | 7 |  | | |
| **4** | | 8 |  | | |
|  | |  |  | | |
| **Section 1.4 General Risk Factor** | **Yes** | **No** | **General Risk Factor** | **Yes** | **No** |
| Previous cellulitis | * |  | Previous DVT |  |  |
| **Number of episodes of previous cellulitis* | _____ |  | Diabetes mellitus |  |  |
| **Number of episodes of cellulitis in currently affected area* | _____ |  | Diabetic foot complications |  |  |
| Peripheral arterial disease |  |  | Peripheral neuropathy |  |  |
| Chronic venous disease |  |  | Current intravenous drug use |  |  |
| Previous MRSA colonization |  |  | Previous intravenous drug use |  |  |
| Previous MRSA infection |  |  | Current homelessness |  |  |
| HIV+ |  |  | Eczema |  |  |

### **Section 1.5 Charlson Co-Morbity Index**

Tick the relevant Boxes and add the corresponding number in brackets () to achieve the total score. Note the maximum score is 24

| **Medical History** | **Yes** | **No** | **Medical History** | **Yes** | **No** |
| --- | --- | --- | --- | --- | --- |
| Congestive cardiac failure (2) |  |  | Mild liver disease (2) |  |  |
| Dementia (2) |  |  | Any malignancy including leukemia / lymphoma (2) |  |  |
| Chronic pulmonary disease (1) |  |  | Moderate or severe liver disease (4) |  |  |
| Rheumatological disease (1) |  |  | Metastatic solid tumour (6) |  |  |
| Diabetes with chronic complications (1) |  |  | AIDS / HIV (4) |  |  |
| Hemiplegia or paraplegia (2) |  |  | Renal disease (1) |  |  |
| **Total score: _________ maximum score = 24** | | | | | |

| **Drug Name**  **(Generic)** | **Indication** | **Dose/**  **Units** | **Route** | **Frequency** | **Start Date**  ***(DD-MM-YYYY)*** | **Stop Date**  ***(DD-MM-YYYY)*** | **✓If Ongoing At Visit** |
| --- | --- | --- | --- | --- | --- | --- | --- |
|  |  |  |  |  | **____/_____/_____** | **____/_____/_____** |  |
|  |  |  |  |  | **____/_____/_____** | **____/_____/_____** |  |
|  |  |  |  |  | **____/_____/_____** | **____/_____/_____** |  |
|  |  |  |  |  | **____/_____/_____** | **____/_____/_____** |  |
|  |  |  |  |  | **____/_____/_____** | **____/_____/_____** |  |
|  |  |  |  |  | **____/_____/_____** | **____/_____/_____** |  |
|  |  |  |  |  | **____/_____/_____** | **____/_____/_____** |  |
|  |  |  |  |  | **____/_____/_____** | **____/_____/_____** |  |
|  |  |  | **Antibiotics** |  |  |  |  |
|  |  |  |  |  | **____/_____/_____** | **____/_____/_____** |  |
|  |  |  |  |  | **____/_____/_____** | **____/_____/_____** |  |
|  |  |  |  |  | **____/_____/_____** | **____/_____/_____** |  |
|  |  |  |  |  | **____/_____/_____** | **____/_____/_____** |  |
|  |  |  |  |  | **____/_____/_____** | **____/_____/_____** |  |

#### **Section 1.6 Current Medication at Baseline**

### **Part B Physical Assessment Of Lesion**

### **Section 1.7 Type of Lesion**

Abscess Cellulitis Infected Wound

### **Section 1.8 Vital Signs**

Blood Pressure ______ /_______ mmHg Heart Rate______/min

Temperature______.______°c SPO2 ________ % Resp. rate ______/min

### **Section 1.9 Location of Lesion**

Mark the Site of Lesion with an X


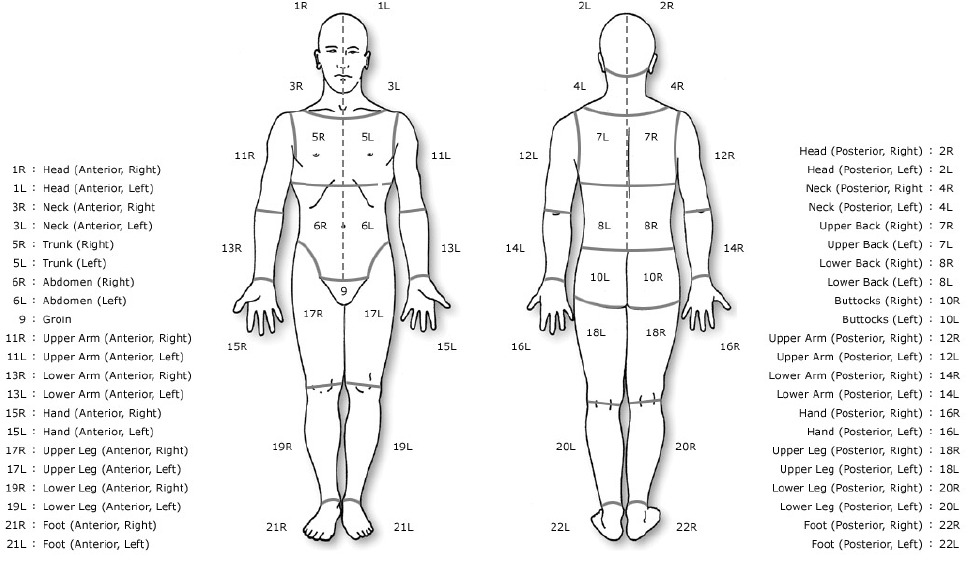


#### **Section 1.10 Lesion Size Characteristics – All Lesion Types**

1. Is erythema present? Yes* No

**If yes what are the maximal dimensions* _______._________ (cm) X _________.________(cm)

**Surface Area** = ___________ **cm^2^**

1. Is swelling/induration present? Yes* No

**If yes what are the maximal dimensions* _______.________(cm) X________.___________(cm)

**Surface Area** = ______________ **cm^2^**

1. Is purulent drainage present? Yes No
2. Is tenderness to palpation present? Yes No

#### **Complete the Following Section for Abscess OR Wound Infection Only**

##### *Section 1.10.1* **ABSCESS***:* complete section for abscess only

Describe the maximal depth of the abscess cavity:

1. Limited to skin/subcutaneous tissues Yes No
2. Involving deep fascia/muscle Yes No

3. Was incision and drainage performed Yes* No

**If yes*

Estimate the maximal length of the abscess cavity _______.________(cm)

Estimate the maximal width of the abscess cavity ________._______(cm)

Estimate the maximal depth of the abscess cavity _______.________(cm)

##### *Section 1.10.2* **WOUND INFECTION***:* complete section for wound infections only

1. Characterize the wound (Tick ☑ one only) Ulcer

Complex/Stellate/Irregular laceration

Linear Laceration

Abrasion

Other, Specify_______________________________

2. What is the maximal wound length? _______.______(cm)

3. Estimate the maximal depth of the wound (Tick ☑ one only)

Limited to skin only

Involves subcutaneous tissue

Involves deep fascia/muscle

4. Did a previous surgical procedure lead to the current infection Yes * No

**If Yes please specify procedure*__________________________________________________

#### **Section 1.11 Local Risk Factor Profile**

| **Ulceration & Venous Disease** | Yes | No |
| --- | --- | --- |
| Venous ulcer |  |  |
| Arterial ulcer |  |  |
| Pressure ulcer |  |  |
| Varicose veins |  |  |
| Venous stasis dermatitis |  |  |
| **Skin Disease** | Yes | No |
| Skin breakdown due to skin condition |  |  |
| **Fungal Foot Disease** | Yes | No |
| Toe web maceration |  |  |
| Toenail dystrophy |  |  |
| **Lymphoedema** | Yes | No |
| Oedema from underlying comorbidity |  |  |
| Lymphoedema |  |  |
| Previous surgery to affected body part |  |  |

#### **Section 1.12 Results of Laboratory Investigations – Complete as applicable**

*Section 1.12.1* Laboratory Swab/Pregnancy Result

1. Microbiological Swab Taken Yes No

2. Pregnancy Result N/A Positive Negative

Complete Section 1.12.1 only if bloods are taken

*Section 1.12.2* Laboratory Blood Results

White Cell Count _________ Neutrophil Count ____________

C Reactive Protein ________ Urea ________ Creatinine _______________

#### **Section 1.13 Participant Eligibility Review/Investigator Sign Off**

| **End of Screening Visit Checklist:** | | Yes | No |
| --- | --- | --- | --- |
| 1. | Does the participant satisfy the inclusion and exclusion criteria to date? |  |  |
| 2. | Have all visit procedures been completed? |  |  |
| 3. | Was the study product administered? *Complete Section 1.14 below |  |  |
| 4. | Was a return appointment scheduled? |  |  |

Is the patient eligible to take part in the clinical trial? Yes No *

**If the patient is deemed a screen failure state reason*

1.____________________________________________

2.____________________________________________

Investigator’s Signature __________________________________ Date ____/______/________

#### **Section 1.14 Participant Randomisation/ Drug Dispensing**

| Penicillin V Med I.D Number |  |
| --- | --- |
| Date of dosing (dd/mm/yyyy) | ____/_____/_____ |
| Time of Dosing (24hour) | ____ ____:____ ____ |

##### **Section 1.14.1 Beaumont Sub-Study Only - MEMS® Cap Administration**

| Mems Cap Device Serial Number | Date of Activation  (dd/mm/yyyy) | Time of Activation  (24 hr) |
| --- | --- | --- |
|  | ____/_____/______ | ____ ____:____ ____ |

**STUDY VISIT COMPLETE**

Name of study staff completing **Baseline** visit (*Signature*):_____________________________________


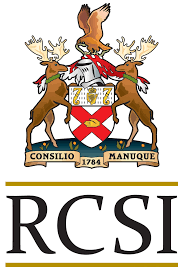


**eARLY cLINICAL RESPONSE VISIT (DAY 2-3)**

**CASE Report FORM**

| **Participant Details** | |
| --- | --- |
| Patient I.D |  |
| Site I.D |  |
| Date of Visit |  |
| Time of Visit (24hour format) |  |

Name of study staff completing **ECR** visit section (**Signature**):___________________________________

#### **Section 2.0 Type of Contact Visit**

#### Sections relevant to phone call visits are shaded in grey throughout this form.

**VISIT TYPE** 1. In-Person Visit 2. Phone Call* 3. DNA*

**** Reason for*** DNA and Phone Call Visits Only

| Subjects Illness or injury Subject Forgot  Subject refusal Site decision/error  Transport problems Unable to contact  Scheduling difficulties temporarily out of area  Other specify ___________________________ |
| --- |

## **Section 2.1 HRQL and Health Resource Use Questionnaires**

Has the patient completed the questionnaires? *If no state reason in comment section*

1. EQ-5D-5L Yes No
2. SF 12 Yes No
3. ESTI-Score Yes No
4. Health Resource Use Questionnaire Yes No

Comment______________________________________________________________________________________

## **Section 2.2 Vital Signs**

Blood Pressure _____ /_______ mmHg Heart Rate_________/min

Temperature___.___°c SPO2 ________ % Resp. rate ________/min

### **Section 2.3 Current Lesion Size Characteristics –All Lesion types**

1. Is erythema present? Yes* No

**If yes what is the maximal dimensions* ______. _______(cm) X _______. _______(cm)

**Surface area = ____________cm^2^**

1. Is swelling/induration present? Yes* No

**If yes what is the maximal dimensions* ______. _______(cm) X _______.________(cm)

**Surface area = ____________ cm^2^**

#### ***COMPLETE THE FOLLOWING SECTION FOR ABSCESS OR WOUND INFECTION ONLY***

##### Section 2.4.1 **ABSCESS***:* complete section for abscess only

Describe the maximal depth of the abscess cavity:

1. Limited to skin/subcutaneous tissues Yes No
2. Involve deep fascia/muscle Yes No

3. Was incision and drainage performed Yes* No

**If yes*

*Estimate the maximal length of the abscess cavity* _______.______(cm)

*Estimate the maximal width of the abscess cavity* _______.______(cm)

*Estimate the maximal depth of the abscess cavity* _______.______(cm)

##### Section 2.4.2 **WOUND INFECTION***:* complete section for wound infections only

1. Characterise the wound (Tick ☑ one only) Ulcer

Complex/Stellate Irregular laceration

Linear Laceration

Abrasion

Other, Specify_______________________________

2. What is the maximal wound length? _______.______(cm)

3. Estimate the maximal depth of the wound (Tick ☑ one only)

Limited to skin

Involves Subcutaneous Tissue

Involves deep fascia/muscle

4. Did a previous surgical procedure lead to the current infection Yes No

#### **Section 3.4 Medication Adherence**

Has the participant taken all of their antibiotic doses as prescribed? Yes No *

*If No how many times would the participant estimate they did not take their doses as prescribed? (*estimate*) _________

When did the participant stop? (*circle one*) Day 1 2 3

Reason?_______________________________________________________________________________

Any times the MEMS cap was opened and a dose not taken? (Approximate Time)

Date ____/_____/_____ Times ____:_____

Date ____/_____/_____ Times ____:_____

#### **Section 2.6 Clinical Failure Assessment**

| **1.** Is there an increase in surface area of either erythema and/or induration ≥ 20% from baseline visit? Yes No (*Refer to surface area on page 11)*  **2**. Is there a worsening of any of the following clinical features such that in your clinical judgement a change in either of the prescribed antibiotics is necessary:  1. Is there increased swelling Yes No  2 Is there an increased purulent drainage? Yes No  3. Is there increased warmth? Yes No  4. Is there increased tenderness? Yes No  **Telephone Visit Only**   1. Has the swelling Increased Decreased Same 2. Has the redness Increased Decreased Same 3. Has the tenderness Increased Decreased Same 4. Has the pain Increased Decreased Same   **3.** Based on clinical judgement and from the above answers, is the patient a clinical failure?  Yes No |
| --- |

**N.B If the visit is conducted by telephone and if the subject is a clinical failure the study investigator should instruct the subject to return to the E.D and schedule a time for an in-person assessment.**

**COMPLETE THE FOLLOWING SECTION ONLY IF THE PATIENT IS DEEMED A CLINICAL FAILURE**

#### **SECTION 2.6.1 CLINICAL FAILURE**

Was drainage present (either spontaneous or drainage after I&D) Yes* No

*If Yes*

Was the drainage purulent? Yes No

Was a wound culture obtained? Yes* No

(*Required if subject is a clinical failure and there is drainage material*)

#### **If wound culture obtained what was the method used*?

#### Swab of drainage

#### Needle aspiration of Abscess

Other, Specify _______________________

#### **Rescue Therapy**

Was the subject admitted to the hospital for in-hospital drainage or to receive IV antibiotics at this visit? Yes No

Did the subject develop an invasive infection since the previous baseline visit? Yes No

If Yes, did the subject develop:

Severe sepsis/septic shock? Yes No

Endocarditis Yes No

Pneumonia Yes No

Necrotizing soft tissue? Yes No

Osteomyelitis Yes No

Bacteremia Yes No

Other, Specify _________________________________________________________________

#### **Section 2.7 Concomitant Medications and Adverse Event**

| Does the researcher consider there to be any signs of an adverse event? *if Yes, please complete an AE form (Appendix 1) | Yes * | No |
| --- | --- | --- |
| Did the patient report taking any concomitant medication since the last visit? *if Yes please complete the concomitant medication form (Appendix 2) | Yes * | No |

Signature of Study Staff that completed this form __________________________________________________

Date _____/________/_________

**NOTE**; **INFORM PATIENT TO BRING THEIR USED AND UNUSED STUDY MEDICATION FOR THEIR follow up VISIT**


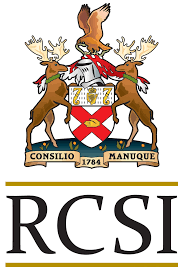


**END OF TREATMENT VISIT (dAY 8-10)**

**CASE Report FORM**

| **Participant Details** | |
| --- | --- |
| Patient I.D |  |
| Site I.D |  |
| Date of Visit |  |
| Time of Visit (24hour format) |  |

Name of study staff completing **EOT** visit (**Signature**):_______________________________________________________

#### **SECTION 3.0 Type of Contact Visit**

#### *Sections relevant to phone call visits are shaded in grey throughout this form.*

**VISIT TYPE** 1. In-Person Visit 2. Phone Call* 3. DNA*

**** Reason For DNA And Phone Call Visits Only***

| Subjects Illness or injury Subject Forgot  Subject refusal Site decision/error  Transport problems Unable to contact  Scheduling difficulties Temporarily out of area  Other specify ___________________________ |
| --- |

## **Section 3.1 HRQL and Health Resource Use Questionnaires**

Has the patient completed the questionnaires? *If no state reason below*

1. EQ-5D-5L Yes No
2. SF 12 Yes No
3. ESTI-Score Yes No
4. Health Resource Use Questionnaire Yes No

Reason______________________________________________________________________________________

## **Section 3.2 Vital Signs**

Blood Pressure _____ /_______ mmHg Heart Rate_________/min

Temperature___.___°c SPO2 ________ % Resp. rate ________/min

#### **Section 3.3 Current Lesion Size Characteristics – All Lesion Types**

1. Is erythema present? Yes* No

**If yes what is the maximal dimensions* ______. _______(cm) X _______. _______(cm)

**Surface area = ____________cm^2^**

1. Is swelling/induration present? Yes* No

**If yes what is the maximal dimensions* ______. _______(cm) X _______.________(cm)

**Surface area = ____________ cm^2^**

#### ***COMPLETE THE FOLLOWING SECTION FOR ABSCESS OR WOUND INFECTION ONLY***

##### Section 3.3.1 **ABSCESS**: complete section for abscess only

Describe the maximal depth of the abscess cavity:

1. Limited to skin/subcutaneous tissues Yes No
2. Involve deep fascia/muscle Yes No

3. Was incision and drainage performed Yes* No

**If yes*

*Estimate the maximal length of the abscess cavity* _______.______(cm)

*Estimate the maximal width of the abscess cavity* _______.______(cm)

*Estimate the maximal depth of the abscess cavity* _______.______(cm)

##### Section 3.3.2 **WOUND INFECTION***:* complete section for wound infections only

Characterise the wound (Tick ☑ one only) Ulcer

Complex/Stellate Irregular laceration

Linear Laceration

Abrasion

Other, Specify_______________________________

What is the maximal wound length? _______.______(cm)

Estimate the maximal depth of the wound (Tick ☑ one only)

Limited to skin

Involves Subcutaneous Tissue

Involves deep fascia/muscle

Did a previous surgical procedure lead to the current infection Yes No

#### **Section 3.4 Medication Adherence**

Has the participant taken all of their antibiotic doses as prescribed? Yes No *

*If No how many times would the participant estimate they did not take their doses as prescribed? (*estimate*) _________

When did the participant stop? (*circle one*) Day 1 2 3 4 5 6 7

Reason?_______________________________________________________________________________

Has the patient returned their used and unused study medication? Yes No

**Pill count**: Number of pills remaining ___________

How many times was the MEMs Cap opened and a dose not taken (Approximate time)?

Date ____/____/_____ Time _____/_____/_____

#### **Section 3.5 Clinical Failure Assessment**

1. Is there an increase in surface area of either erythema and/or induration ≥ 20% from baseline visit? Yes No (*Refer to surface area on page 11)*

2. Is there a worsening of any of the following clinical features such that in your clinical judgement a change in either of the prescribed antibiotics is necessary?

1. Is there increased swelling Yes No

2. Is there an increased purulent drainage? Yes No

3. Is there increased warmth? Yes No

4. Is there increased tenderness? Yes No

Telephone Visit Only

1. Has the swelling Increased Decreased Same
2. Has the redness Increased Decreased Same
3. Has the tenderness Increased Decreased Same
4. Has the pain Increased Decreased Same

Based on clinical judgement and from the above answers, is the patient a clinical failure?

Yes No*

***N.B If the visit is conducted by telephone and if the subject is a clinical failure the study investigator should instruct the subject to return to the E.D and schedule a time for an in-person assessment.**

***COMPLETE THE FOLLOWING SECTION ONLY IF THE PATIENT IS DEEMED A CLINICAL FAILURE**

#### **Section 3.5.1 Clinical Failure**

Was drainage present (either spontaneous or drainage after I&D) Yes* No

**If Yes*

Was the drainage purulent? Yes No

Was a wound culture obtained? Yes* No

(*Required if subject is a clinical failure and there is drainage material*)

#### **If wound culture obtained what was the method used*?

#### Swab of drainage

#### Needle aspiration of Abscess

Other, Specify _______________________

#### **Rescue Therapy**

Was the subject admitted to the hospital for in-hospital drainage or to receive IV antibiotics at this visit? Yes No

Did the subject develop an invasive infection since the previous baseline visit? Yes No

If Yes, did the subject develop:

Severe sepsis/septic shock? Yes No

Endocarditis Yes No

Pneumonia Yes No

Necrotizing soft tissue? Yes No

Osteomyelitis Yes No

Bacteremia Yes No

Other, Specify _________________________________________________________________

#### **Section 3.6. MEMS® Study for Beaumont Study Site Only**

1. Has the MEMS® cap been downloaded for patients enrolled in adherence sub study? Yes No

#### **Section 3.7 Concomitant Medications and Adverse Event**

| Does the researcher consider there to be any signs of an adverse event?  *if Yes, please complete an AE form | Yes * | No |
| --- | --- | --- |
| Did the patient report taking any concomitant medication since the last visit? *if Yes please complete the concomitant medication form (Appendix 2) | Yes * | No |

Signature of Study Staff that completed this form __________________________________________________

Date ___/________/_________

Signature of Study Staff that made changes to this form_________________________________________________

Date ___/________/_________

**NOTE**; **INFORM PATIENT TO BRING THEIR USED AND UNUSED STUDY MEDICATION FOR THEIR follow up VISIT**


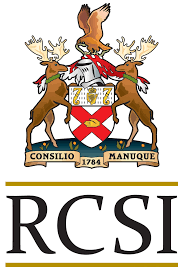


**TEST OF CURE VISIT (DAY 14-21)**

**CASE Report FORM**

| **PARTICIPANT detailS** | |
| --- | --- |
| Patient I.D |  |
| Site I.D |  |
| Date of Visit |  |
| Time of Visit (24hour format) |  |

Name of study staff completing **TOC** visit section (**Signature**):_______________________________________________________

#### **Section 4.0 Type of Contact Visit**

#### *Sections relevant to phone call visits are shaded in grey throughout this form.*

**VISIT TYPE** 1. In-Person Visit 2. Phone Call* 3. DNA*

**** Reason for DNA and Phone Call Visits Only***

| Subjects Illness or injury Subject Forgot  Subject refusal Site decision/error  Transport problems Unable to contact  Scheduling difficulties Temporarily out of area  Other specify ___________________________ |
| --- |

#### **section 4.1 HRQL And Health Resource Use Questionnaires**

Has the patient completed the questionnaires? *If no state reason in comment section*

1. EQ-5D-5L Yes No
2. SF 12 Yes No
3. ESTI-Score Yes No
4. Health Resource Use Questionnaire Yes No

Comment_________________________________________________________________________________

#### **Section 4.2 Vital Signs**

Blood Pressure _____ /_______ mmHg Heart Rate_________/min

Temperature_______.______°c SPO2 ________ % Resp. rate ________/min

#### **Section 4.3 Current Lesion Size Characteristics**

1. Is erythema present? Yes* No

**If yes what were the maximal dimensions* _______. _______(cm) X_______.________(cm)

Surface Area __________ cm^2^

1. Is swelling/induration present? Yes* No

**If yes what were the maximal dimensions* _______.________(cm) X_______. _______(cm)

Surface Area ___________ cm^2^

#### **COMPLETE THE FOLLOWING SECTION FOR ABSCESS OR WOUND INFECTION ONLY**

##### Section 4.3.1 **ABSCESS**: complete section for abscess only

Describe the maximal depth of the abscess cavity:

1. Limited to skin/subcutaneous tissues Yes No
2. Involve deep fascia/muscle Yes No

3. Was incision and drainage performed Yes* No

**If yes*

*Estimate the maximal length of the abscess cavity* _______.______(cm)

*Estimate the maximal width of the abscess cavity* _______.______(cm)

*Estimate the maximal depth of the abscess cavity* _______.______(cm)

##### Section 4.3.2 **WOUND INFECTION***:* complete section for wound infections only

Characterise the wound (Tick ☑ one only) Ulcer

Complex/Stellate Irregular laceration

Linear Laceration

Abrasion

Other, Specify_______________________________

What is the maximal wound length? _______.______(cm)

Estimate the maximal depth of the wound (Tick ☑ one only)

Limited to skin

Involves Subcutaneous Tissue

Involves deep fascia/muscle

Did a previous surgical procedure lead to the current infection Yes No

**Section 4.5 Clinical Failure Assessment**

1. Is there an increase in surface area of either erythema and/or induration ≥ 20% from baseline visit? Yes No (*Refer to surface area on page 11)*

2. Is there a worsening of any of the following clinical features such that in your clinical judgement a change in either of the prescribed antibiotics is necessary?

1. Is there increased swelling Yes No

2. Is there an increased purulent drainage? Yes No

3. Is there increased warmth? Yes No

4. Is there increased tenderness? Yes No

**Telephone Visit Only**

1. Has the swelling Increased Decreased Same

2. Has the redness Increased Decreased Same

3. Has the tenderness Increased Decreased Same

4. Has the pain Increased Decreased Same

Based on clinical judgement and from the above answers, is the patient a clinical failure?

Yes No*

***N.B If the visit is conducted by telephone and if the subject is a clinical failure the study investigator should instruct the subject to return to the E.D and schedule a time for an in-person assessment.**

***COMPLETE THE FOLLOWING SECTION ONLY IF THE PATIENT IS DEEMED A CLINICAL FAILURE**

#### ***Section 4.5.1 Clinical Failure***

Was drainage present (either spontaneous or drainage after I&D) Yes* No

**If Yes*

Was the drainage purulent? Yes No

Was a wound culture obtained? Yes* No

(*Required if subject is a clinical failure and there is drainage material*)

#### **If wound culture obtained what was the method used*?

#### Swab of drainage

#### Needle aspiration of Abscess

Other, Specify _______________________

#### **Rescue Therapy**

Was the subject admitted to the hospital for in-hospital drainage or to receive IV antibiotics at this visit? Yes No

Did the subject develop an invasive infection since the previous baseline visit? Yes No

If Yes, did the subject develop:

Severe sepsis/septic shock? Yes No

Endocarditis Yes No

Pneumonia Yes No

Necrotizing soft tissue? Yes No

Osteomyelitis Yes No

Bacteremia Yes No

Other, Specify _________________________________________________________________

#### **Section 4.6 Concomitant Medications and Adverse Event**

| Does the researcher consider there to be any signs of an adverse event? *if Yes, please complete an AE form (Appendix 1) | Yes * | No |
| --- | --- | --- |
| Did the patient report taking any concomitant medication since the last visit? *if Yes please complete the concomitant medication form (Appendix 2) | Yes * | No |

Signature of Study Staff that completed this form __________________________________________________

Date _______/________/_________


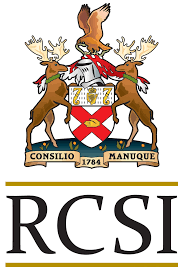


### **conconmitant medication**

**confidential**

INSTRUCTIONS FOR DOCUMENTING CONCOMITANT MEDICATION

- Any medication that the patient is taking other than the study drugs specified in the protocol is considered a concomitant medication.
- All concomitant medications will be recorded
- Document (page) using the generic drug name.
- If the patient is on an **oral contraceptive pill**, they should be advised the use of barrier contraception while they are enrolled in this study.

| Drug Name  (Generic) | Indication | Dose | Route | Frequency | Start date  (dd-mm-yyyy) | Stop date  (dd-mm-yyyy) | ✓if ongoing at visit |
| --- | --- | --- | --- | --- | --- | --- | --- |
|  |  |  |  |  | **____/_____/_____** | **____/_____/_____** |  |
|  |  |  |  |  | **____/_____/_____** | **____/_____/_____** |  |
|  |  |  |  |  | **____/_____/_____** | **____/_____/_____** |  |
|  |  |  |  |  | **____/_____/_____** | **____/_____/_____** |  |
|  |  |  |  |  | **____/_____/_____** | **____/_____/_____** |  |
|  |  |  |  |  | **____/_____/_____** | **____/_____/_____** |  |
|  |  |  |  |  | **____/_____/_____** | **____/_____/_____** |  |
|  |  |  |  |  | **____/_____/_____** | **____/_____/_____** |  |
|  |  |  |  |  | **____/_____/_____** | **____/_____/_____** |  |
|  |  |  |  |  | **____/_____/_____** | **____/_____/_____** |  |
|  |  |  |  |  | **____/_____/_____** | **____/_____/_____** |  |
|  |  |  |  |  | **____/_____/_____** | **____/_____/_____** |  |

### Appendix 1 Conconmitant Medication

| Drug Name  (Generic) | Indication | Dose | Route | Frequency | Start Date  (dd-mm-yyyy) | Stop Date  (dd-mm-yyyy) | ✓If ongoing at visit |
| --- | --- | --- | --- | --- | --- | --- | --- |
|  |  |  |  |  | **____/_____/_____** | **____/_____/_____** |  |
|  |  |  |  |  | **____/_____/_____** | **____/_____/_____** |  |
|  |  |  |  |  | **____/_____/_____** | **____/_____/_____** |  |
|  |  |  |  |  | **____/_____/_____** | **____/_____/_____** |  |
|  |  |  |  |  | **____/_____/_____** | **____/_____/_____** |  |
|  |  |  |  |  | **____/_____/_____** | **____/_____/_____** |  |
|  |  |  |  |  | **____/_____/_____** | **____/_____/_____** |  |
|  |  |  |  |  | **____/_____/_____** | **____/_____/_____** |  |
|  |  |  |  |  | **____/_____/_____** | **____/_____/_____** |  |
|  |  |  |  |  | **____/_____/_____** | **____/_____/_____** |  |
|  |  |  |  |  | **____/_____/_____** | **____/_____/_____** |  |
|  |  |  |  |  | **____/_____/_____** | **____/_____/_____** |  |
|  |  |  |  |  | **____/_____/_____** | **____/_____/_____** |  |
