# Supplementary material for: The Penicillin for the Emergency Department Outpatient treatment of CELLulitis (PEDOCELL) trial: update to the study protocol and detailed statistical analysis plan (SAP)
Source: Trials. 2017 Aug 24;18:391. doi: 10.1186/s13063-017-2121-2 (PMC5571617; doi:10.1186/s13063-017-2121-2)
Supplement: Supplementary file 2 — Health-related quality of life (HRQoL) questionnaires. The HRQoL questionnaires to be used in the PEDOCELL trial are to be found in Additional File 2. The EQ-5D-5L, the SF-12 and the Extremity Soft Tissue Infection (ESTI) score will be used to measure HRQoL outcomes in patients enrolled to the PEDOCELL trial at each follow-up visit. (DOCX 86 kb) [file 13063_2017_2121_MOESM2_ESM.docx]

**Health Resource Use Questionnaires**

Baseline Visit 2

Early Clinical Response (ECR) Visit (Day 2-3) 6

End-of-Treatment (EOT) Visit (Day 8-10) 10

Test-of-Cure (TOC) Visit (Day 14-21) 14

Unscheduled Visit 18

# Health Resource Use Questionnaire: Baseline Visit

In order to accurately measure the cost of cellulitis treatments, we would like to know the number of times you have seen a healthcare professional (i.e. a doctor or nurse) in the last 3 months. If the health care you received was related to your cellulitis, record this in the ‘Because of cellulitis’ column. If the health care was for any other reason, enter this in the ‘Other’ column. Please answer every question, even if the answer is ‘0’.

1. **In the past 3 months, how many times have you consulted with any of the following healthcare professionals?**

|  | Because of cellulitis | Other |
| --- | --- | --- |
| General practitioner (GP) at your GP’s surgery |  |  |
| GP in your home |  |  |
| A **nurse** at your GP’s surgery |  |  |
| A **nurse** in your house |  |  |
| A **doctor** in a hospital or other location |  |  |
| A **nurse** in a hospital or other location |  |  |

1. **In the past 3 months, how many times have you:**

|  | Because of cellulitis | Other |
| --- | --- | --- |
| Been admitted to hospital **without** staying overnight (i.e. for a minor procedure or day surgery)? |  |  |
| Been admitted to hospital as an in-patient (i.e. stayed for 1 or more nights)? |  |  |
| If, since the last time you were assessed for this trial, you have been a hospital in-patient, please record how many nights you stayed in hospital. If you have stayed in hospital more than once please add the nights you stayed for each visit together and record the total. |  |  |

|  | **Patient*** |
| --- | --- |
| How did you get to the hospital today?  **CODE:** 1. Walk; 2. Car; 3. Public transport; 4. Taxi; 5. Other. |  |
| How much did the trip cost? | N/A **or** € |
| If you had other expenses (e.g. baby sitter), how much was this? | N/A **or** € |
| Is your travelling time and cost likely to be the same in the future? | YES NO |
| If **NO**, describe briefly how  the arrangements might be different… |  |

1. **Travel to hospital:**
2. **Work Impacts of Cellulitis**

Are you 1. Employed full-time  5. A housewife/husband

***(tick one box)*** 2. Employed part-time  6. Retired

3. Unemployed  7. Other (Describe.…)

4. A student

**If unemployed:**

a) Month / year last in paid employment  /

*m m y y*

b) Job title of your last paid job

c) Did you give up work because of your cellulitis? Yes  No

**If employed:**

a) What is your job title?

b) How many hours do you usually work each week

c) How many days have you been absent from work since the last assessment for this trial?

d) Of these days absent, how many were due to cellulitis?

e) Has your cellulitis affected your working hours? Yes  No

If **YES**: How many hours less have you worked per day?

1. **Medications**

Please tell us about medications and over-the-counter treatments you have used in the past 3 months:

Medication type –

Dose-

Frequency -

Who pays for the treatments (pharmaceutical and clinic time)?

(***tick one box***)

1. Patient
2. Insurance
3. Spouse
4. Parent/ guardian
5. Child
6. Other

**6. Health Insurance**

How much a year do you spend on medical/ health insurance?

Do you have a medical card? Yes  No

# Health Resource Use Questionnaire:

# Early Clinical Response (ECR) Visit (Day 2-3)

In order to accurately measure the cost of cellulitis treatments, we would like to know the number of times you have seen a healthcare professional (i.e. a doctor or nurse). If the health care you received was related to your cellulitis, record this in the ‘Because of cellulitis’ column. If the health care was for any other reason, enter this in the ‘Other’ column. Please answer every question, even if the answer is ‘0’.

1. **Since you were enrolled in this trial, how many times have you consulted with any of the following healthcare professionals?**

|  | Because of cellulitis | Other |
| --- | --- | --- |
| General practitioner (GP) at your GP’s surgery |  |  |
| GP in your home |  |  |
| A **nurse** at your GP’s surgery |  |  |
| A **nurse** in your house |  |  |
| A **doctor** in a hospital or other location |  |  |
| A **nurse** in a hospital or other location |  |  |

1. **Since you were enrolled in this trial, how many times have you:**

|  | Because of cellulitis | Other |
| --- | --- | --- |
| Been admitted to hospital **without** staying overnight (i.e. for a minor procedure or day surgery)? |  |  |
| Been admitted to hospital as an in-patient (i.e. stayed for 1 or more nights)? |  |  |
| If, since the last time you were assessed for this trial, you have been a hospital in-patient, please record how many nights you stayed in hospital. If you have stayed in hospital more than once please add the nights you stayed for each visit together and record the total. |  |  |

1. **Travel to hospital:**

|  | **Patient*** |
| --- | --- |
| How did you get to the hospital today?  **CODE:** 1. Walk; 2. Car; 3. Public transport; 4. Taxi; 5. Other. |  |
| How much did the trip cost? | N/A **or** € |
| If you had other expenses (e.g. baby sitter), how much was this? | N/A **or** € |
| Is your travelling time and cost likely to be the same in the future? | YES NO |
| If **NO**, describe briefly how  the arrangements might be different… |  |

1. **Work Impacts of Cellulitis**

Are you 1. Employed full-time  5. A housewife/husband

***(tick one box)*** 2. Employed part-time  6. Retired

3. Unemployed  7. Other (Describe.…)

4. A student

**If unemployed:**

a) Month / year last in paid employment  /

*m m y y*

b) Job title of your last paid job

c) Did you give up work because of your cellulitis? Yes  No

**If employed:**

a) What is your job title?

b) How many hours do you usually work each week

c) How many days have you been absent from work since you were enrolled in this trial?

d) Of these days absent, how many were due to cellulitis?

e) Has your cellulitis affected your working hours since you were enrolled in this trial? Yes  No

If **YES**: How many hours less have you worked per day?

How many hours less have you worked per week?

How often does your cellulitis affect your work?

***(tick one box)***

1. Once or twice a day

2. Once or twice a week

1. **Medications**

Please tell us about medications and over-the-counter treatments you have used since you were enrolled in this trial.

Medication type –

Dose-

Frequency -

Who pays for the treatments (pharmaceutical and clinic time)?

(***tick one box***)

1. Patient
2. Insurance
3. Spouse
4. Parent/ guardian
5. Child
6. Other
7. **Health Insurance**

How much a year do you spend on medical/ health insurance?

___________________________________

Do you have a medical card? Yes  No

# Health Resource Use Questionnaire:

# End-of-Treatment (EOT) Visit (Day 8-10)

In order to accurately measure the cost of cellulitis treatments, we would like to know the number of times you have seen a healthcare professional (i.e. a doctor or nurse). If the health care you received was related to your cellulitis, record this in the ‘Because of cellulitis’ column. If the health care was for any other reason, enter this in the ‘Other’ column. Please answer every question, even if the answer is ‘0’.

1. **Since the last time you were assessed for this trial, how many times have you consulted with any of the following healthcare professionals?**

|  | Because of cellulitis | Other |
| --- | --- | --- |
| General practitioner (GP) at your GP’s surgery |  |  |
| GP in your home |  |  |
| A **nurse** at your GP’s surgery |  |  |
| A **nurse** in your house |  |  |
| A **doctor** in a hospital or other location |  |  |
| A **nurse** in a hospital or other location |  |  |

1. **Since the last time you were assessed for this trial, how many times have you:**

|  | Because of cellulitis | Other |
| --- | --- | --- |
| Been admitted to hospital **without** staying overnight (i.e. for a minor procedure or day surgery)? |  |  |
| Been admitted to hospital as an in-patient (i.e. stayed for 1 or more nights)? |  |  |
| If, since the last time you were assessed for this trial, you have been a hospital in-patient, please record how many nights you stayed in hospital. If you have stayed in hospital more than once please add the nights you stayed for each visit together and record the total. |  |  |

1. **Travel to hospital:**

|  | **Patient*** |
| --- | --- |
| How did you get to the hospital today?  **CODE:** 1. Walk; 2. Car; 3. Public transport; 4. Taxi; 5. Other. |  |
| How much did the trip cost? | N/A **or** € |
| If you had other expenses (e.g. baby sitter), how much was this? | N/A **or** € |
| Is your travelling time and cost likely to be the same in the future? | YES NO |
| If **NO**, describe briefly how  the arrangements might be different… |  |

1. **Work Impacts of Cellulitis**

Are you 1. Employed full-time  5. A housewife/husband

***(tick one box)*** 2. Employed part-time  6. Retired

3. Unemployed  7. Other (Describe.…)

4. A student

**If unemployed:**

a) Month / year last in paid employment  /

*m m y y*

b) Job title of your last paid job

c) Did you give up work because of your cellulitis? Yes  No

**If employed:**

a) What is your job title?

b) How many hours do you usually work each week

c) How many days have you been absent from work since the last assessment for this trial?

d) Of these days absent, how many were due to cellulitis?

e) Has your cellulitis affected your working hours since the last assessment for this trial? Yes  No

If **YES**: How many hours less have you worked per day?

How many hours less have you worked per week?

How often does your cellulitis affect your work?

***(tick one box)***

1. Once or twice a day

2. Once or twice a week

1. **Medications**

Please tell us about medications and over-the-counter treatments you have used since the last time you were assessed for this trial.

Medication type –

Dose-

Frequency -

Who pays for the treatments (pharmaceutical and clinic time)?

(***tick one box***)

1. Patient
2. Insurance
3. Spouse
4. Parent/ guardian
5. Child
6. Other
7. **Health Insurance**

How much a year do you spend on medical/ health insurance?

___________________________________

Do you have a medical card? Yes  No

# Health Resource Use Questionnaire:

# Test-of-Cure (TOC) Visit (Day 14-21)

In order to accurately measure the cost of cellulitis treatments, we would like to know the number of times you have seen a healthcare professional (i.e. a doctor or nurse). If the health care you received was related to your cellulitis, record this in the ‘Because of cellulitis’ column. If the health care was for any other reason, enter this in the ‘Other’ column. Please answer every question, even if the answer is ‘0’.

1. **Since the last time you were assessed for this trial, how many times have you consulted with any of the following healthcare professionals?**

|  | Because of cellulitis | Other |
| --- | --- | --- |
| General practitioner (GP) at your GP’s surgery |  |  |
| GP in your home |  |  |
| A **nurse** at your GP’s surgery |  |  |
| A **nurse** in your house |  |  |
| A **doctor** in a hospital or other location |  |  |
| A **nurse** in a hospital or other location |  |  |

1. **Since the last time you were assessed for this trial, how many times have you:**

|  | Because of cellulitis | Other |
| --- | --- | --- |
| Been admitted to hospital **without** staying overnight (i.e. for a minor procedure or day surgery)? |  |  |
| Been admitted to hospital as an in-patient (i.e. stayed for 1 or more nights)? |  |  |
| If, since the last time you were assessed for this trial, you have been a hospital in-patient, please record how many nights you stayed in hospital. If you have stayed in hospital more than once please add the nights you stayed for each visit together and record the total. |  |  |

1. **Travel to hospital:**

|  | **Patient*** |
| --- | --- |
| How did you get to the hospital today?  **CODE:** 1. Walk; 2. Car; 3. Public transport; 4. Taxi; 5. Other. |  |
| How much did the trip cost? | N/A **or** € |
| If you had other expenses (e.g. baby sitter), how much was this? | N/A **or** € |
| Is your travelling time and cost likely to be the same in the future? | YES NO |
| If **NO**, describe briefly how  the arrangements might be different… |  |

1. **Work Impacts of Cellulitis**

Are you 1. Employed full-time  5. A housewife/husband

***(tick one box)*** 2. Employed part-time  6. Retired

3. Unemployed  7. Other (Describe.…)

4. A student

**If unemployed:**

a) Month / year last in paid employment  /

*m m y y*

b) Job title of your last paid job

c) Did you give up work because of your cellulitis? Yes  No

**If employed:**

a) What is your job title?

b) How many hours do you usually work each week

c) How many days have you been absent from work since the last assessment for this trial?

d) Of these days absent, how many were due to cellulitis?

e) Has your cellulitis affected your working hours since the last assessment for this trial? Yes  No

If **YES**: How many hours less have you worked per day?

How many hours less have you worked per week?

How often does your cellulitis affect your work?

1. Once or twice a day

2. Once or twice a week

1. **Medications**

Please tell us about medications and over-the-counter treatments you have used since the last time you were assessed for this trial.

Medication type –

Dose-

Frequency -

Who pays for the treatments (pharmaceutical and clinic time)?

(***tick one box***)

1. Patient
2. Insurance
3. Spouse
4. Parent/ guardian
5. Child
6. Other
7. **Health Insurance**

How much a year do you spend on medical/ health insurance?

___________________________________

Do you have a medical card? Yes  No

# Health Resource Use Questionnaire:

# Unscheduled Visit

In order to accurately measure the cost of cellulitis treatments, we would like to know the number of times you have seen a healthcare professional (i.e. a doctor or nurse). If the health care you received was related to your cellulitis, record this in the ‘Because of cellulitis’ column. If the health care was for any other reason, enter this in the ‘Other’ column. Please answer every question, even if the answer is ‘0’.

1. **Since the last time you were assessed for this trial, how many times have you consulted with any of the following healthcare professionals?**

|  | Because of cellulitis | Other |
| --- | --- | --- |
| General practitioner (GP) at your GP’s surgery |  |  |
| GP in your home |  |  |
| A **nurse** at your GP’s surgery |  |  |
| A **nurse** in your house |  |  |
| A **doctor** in a hospital or other location |  |  |
| A **nurse** in a hospital or other location |  |  |

1. **Since the last time you were assessed for this trial, how many times have you:**

|  | Because of cellulitis | Other |
| --- | --- | --- |
| Been admitted to hospital **without** staying overnight (i.e. for a minor procedure or day surgery)? |  |  |
| Been admitted to hospital as an in-patient (i.e. stayed for 1 or more nights)? |  |  |
| If, since the last time you were assessed for this trial, you have been a hospital in-patient, please record how many nights you stayed in hospital. If you have stayed in hospital more than once please add the nights you stayed for each visit together and record the total. |  |  |

1. **Travel to hospital:**

|  | **Patient*** |
| --- | --- |
| How did you get to the hospital today?  **CODE:** 1. Walk; 2. Car; 3. Public transport; 4. Taxi; 5. Other. |  |
| How much did the trip cost? | N/A **or** € |
| If you had other expenses (e.g. baby sitter), how much was this? | N/A **or** € |
| Is your travelling time and cost likely to be the same in the future? | YES NO |
| If **NO**, describe briefly how  the arrangements might be different… |  |

1. **Work Impacts of Cellulitis**

Are you 1. Employed full-time  5. A housewife/husband

***(tick one box)*** 2. Employed part-time  6. Retired

3. Unemployed  7. Other (Describe.…)

4. A student

**If unemployed:**

a) Month / year last in paid employment  /

*m m y y*

b) Job title of your last paid job

c) Did you give up work because of your cellulitis? Yes  No

**If employed:**

a) What is your job title?

b) How many hours do you usually work each week

c) How many days have you been absent from work since the last assessment for this trial?

d) Of these days absent, how many were due to cellulitis?

e) Has your cellulitis affected your working hours since the last assessment for this trial? Yes  No

If **YES**: How many hours less have you worked per day?

How many hours less have you worked per week?

How often does your cellulitis affect your work?

***(tick one box)***

1. Once or twice a day

2. Once or twice a week

1. **Medications**

Please tell us about medications and over-the-counter treatments you have used since the last time you were assessed for this trial.

Medication type –

Dose-

Frequency -

Who pays for the treatments (pharmaceutical and clinic time)?

(***tick one box***)

1. Patient
2. Insurance
3. Spouse
4. Parent/ guardian
5. Child
6. Other
7. **Health Insurance**

How much a year do you spend on medical/ health insurance?

Do you have a medical card? Yes  No
