# Supplementary material for: The Penicillin for the Emergency Department Outpatient treatment of CELLulitis (PEDOCELL) trial: update to the study protocol and detailed statistical analysis plan (SAP)
Source: Trials. 2017 Aug 24;18:391. doi: 10.1186/s13063-017-2121-2 (PMC5571617; doi:10.1186/s13063-017-2121-2)
Supplement: Supplementary file 3 — Health resource use questionnaires. The health resource use questionnaires will be completed at the baseline visit, early clinical response visit, end of treatment visit, test of cure visit and any unscheduled visit. The results of these questionnaires will contribute to the pharmacoeconomic analysis for the PEDOCELL trial. (DOCX 123 kb) [file 13063_2017_2121_MOESM3_ESM.docx]

**Health-Related Quality of Life (HRQoL) Questionnaires**

**PEDOCELL Trial**

**Table of Contents**

EQ-5D-5L Health Questionnaire 2

SF-12^®^ Patient Questionnaire 5

Extremity Soft Tissue Infection (ESTI)-Score 9

|  |
| --- |

| EQ-5D-5L Health Questionnaire |
| --- |
|  |
|  |
|  |

| Under each heading, please tick the ONE box that best describes your health TODAY. | |
| --- | --- |
| **MOBILITY** |  |
| I have no problems in walking about | ❑ |
| I have slight problems in walking about | ❑ |
| I have moderate problems in walking about | ❑ |
| I have severe problems in walking about | ❑ |
| I am unable to walk about | ❑ |
| **SELF-CARE** |  |
| I have no problems washing or dressing myself | ❑ |
| I have slight problems washing or dressing myself | ❑ |
| I have moderate problems washing or dressing myself | ❑ |
| I have severe problems washing or dressing myself | ❑ |
| I am unable to wash or dress myself | ❑ |
| **USUAL ACTIVITIES** *(e.g. work, study, housework, family or leisure activities)* |  |
| I have no problems doing my usual activities | ❑ |
| I have slight problems doing my usual activities | ❑ |
| I have moderate problems doing my usual activities | ❑ |
| I have severe problems doing my usual activities | ❑ |
| I am unable to do my usual activities | ❑ |
| **PAIN / DISCOMFORT** |  |
| I have no pain or discomfort | ❑ |
| I have slight pain or discomfort | ❑ |
| I have moderate pain or discomfort | ❑ |
| I have severe pain or discomfort | ❑ |
| I have extreme pain or discomfort | ❑ |
| **ANXIETY / DEPRESSION** |  |
| I am not anxious or depressed | ❑ |
| I am slightly anxious or depressed | ❑ |
| I am moderately anxious or depressed | ❑ |
| I am severely anxious or depressed | ❑ |
| I am extremely anxious or depressed | ❑ |

The best health you can imagine

| We would like to know how good or bad your health is TODAY. |
| --- |
| This scale is numbered from 0 to 100. |
| 100 means the best health you can imagine. 0 means the worst health you can imagine. |
| Mark an X on the scale to indicate how your health is TODAY. |
| Now, please write the number you marked on the scale in the box below. |

YOUR HEALTH TODAY =

10

0

20

30

40

50

60

80

70

90

100

5

15

25

35

45

55

75

65

85

95

The worst health you can imagine

# SF-12^®^ Patient Questionnaire

**SF**-**12®:**

This information will help your doctors keep track of how you feel and how well you are able to do your usual activities. Answer every question by placing a check mark on the line in front of the appropriate answer. It is not specific for arthritis. If you are unsure about how to answer a question, please give the best answer you can and make a written comment beside your answer.

**1. In general**, would you say your health is:

_____ Excellent (1)

_____ Very Good (2)

_____ Good (3)

_____ Fair (4)

_____ Poor (5)

The following two questions are about activities you might do during a typical day. Does YOUR

HEALTH NOW LIMIT YOU in these activities? If so, how much?

**2. MODERATE ACTIVITIES**, such as moving a table, pushing a vacuum cleaner, bowling, or playing golf:

_____ Yes, Limited A Lot (1)

_____ Yes, Limited A Little (2)

_____ No, Not Limited At All (3)

**3. Climbing SEVERAL flights of stairs:**

_____ Yes, Limited A Lot (1)

_____ Yes, Limited A Little (2)

_____ No, Not Limited At All (3)

During the PAST 4 WEEKS have you had any of the following problems with your work or other regular activities AS A RESULT OF YOUR PHYSICAL HEALTH?

4. ACCOMPLISHED LESS than you would like:

_____ Yes (1)

_____ No (2)

5. Were limited in the KIND of work or other activities:

_____ Yes (1)

_____ No (2)

**SF**-**12®** Page 2 of 3

**SF-12® Cont’d:**

During the PAST 4 WEEKS, were you limited in the kind of work you do or other regular activities AS A RESULT OF ANY EMOTIONAL PROBLEMS (such as feeling depressed or anxious)?

6. ACCOMPLISHED LESS than you would like:

_____ Yes (1)

_____ No (2)

7. Didn’t do work or other activities as CAREFULLY as usual:

_____ Yes (1)

_____ No (2)

8. During the PAST 4 WEEKS, how much did PAIN interfere with your normal work (including both work outside the home and housework)?

_____ Not At All (1)

_____ A Little Bit (2)

_____ Moderately (3)

_____ Quite A Bit (4)

_____ Extremely (5)

The next three questions are about how you feel and how things have been DURING THE PAST 4

WEEKS. For each question, please give the one answer that comes closest to the way you have been

feeling. How much of the time during the PAST 4 WEEKS –

9. Have you felt calm and peaceful?

_____ All of the Time (1)

_____ Most of the Time (2)

_____ A Good Bit of the Time (3)

_____ Some of the Time (4)

_____ A Little of the Time (5)

_____ None of the Time (6)

**SF-12® Cont’d:**

10. Did you have a lot of energy?

_____ All of the Time (1)

_____ Most of the Time (2)

_____ A Good Bit of the Time (3)

_____ Some of the Time (4)

_____ A Little of the Time (5)

_____ None of the Time (6)

11. Have you felt downhearted and blue?

_____ All of the Time (1)

_____ Most of the Time (2)

_____ A Good Bit of the Time (3)

_____ Some of the Time (4)

_____ A Little of the Time (5)

_____ None of the Time (6)

12. During the PAST 4 WEEKS, how much of the time has your PHYSICAL HEALTH OR EMOTIONAL PROBLEMS interfered with your social activities (like visiting with friends, relatives, etc.)?

_____ All of the Time (1)

_____ Most of the Time (2)

_____ A Good Bit of the Time (3)

_____ Some of the Time (4)

_____ A Little of the Time (5)

_____ None of the Time (6)

SF-12® Health Survey © 1994, 2002 by Medical Outcomes Trust and Quality Metric Incorporated. All Rights Reserved

SF-12® is a registered trademark of Medical Outcomes Trust

# Extremity Soft Tissue Infection (ESTI)-Score

| **Symptoms** | **Extreme**  **(5)** | **Severe**  **(4)** | **Moderate**  **(3)** | **Slight**  **(2)** | **None**  **(1)** |
| --- | --- | --- | --- | --- | --- |
| Pain |  |  |  |  |  |
| Swelling |  |  |  |  |  |
| Trouble moving |  |  |  |  |  |
| Stiffness |  |  |  |  |  |
| Soreness |  |  |  |  |  |
| Pressure |  |  |  |  |  |
| Throbbing |  |  |  |  |  |
| **Daily Functioning** | **Unable to carry out function (5)** | **Severe problems**  **(4)** | **Moderate problems**  **(3)** | **Slight problems**  **(2)** | **No problems**  **(1)** |
| Doing your job |  |  |  |  |  |
| Walking |  |  |  |  |  |
| Bathing |  |  |  |  |  |
| Changing clothes |  |  |  |  |  |
| Earning an income |  |  |  |  |  |
| Exercising |  |  |  |  |  |
| Falling asleep |  |  |  |  |  |
| **Emotional Functioning** | **Extremely**  **(5)** | **Severely**  **(4)** | **Moderately**  **(3)** | **Slightly**  **(2)** | **Not**  **(1)** |
| Frustrated |  |  |  |  |  |
| Disappointed |  |  |  |  |  |
| Annoyed |  |  |  |  |  |
| Exhausted |  |  |  |  |  |
| Inconvenienced |  |  |  |  |  |

| **Social Interactions** | **Strongly Agree**  **(5)** | **Agree**  **(4)** | **Neutral**  **(3)** | **Disagree**  **(2)** | **Strongly Disagree**  **(1)** |
| --- | --- | --- | --- | --- | --- |
| You are inconveniencing your friends and family |  |  |  |  |  |
| **TOTAL SCORE** |  |  |  |  |  |
